# Account of Diseases in an Eastern District of London, from December 20, 1806, to January 20, 1807

**Published:** 1807-02

**Authors:** 


### *Account of Diseases in an Eastern District of London, from December* 20, 1806, *to January* 20, 1807.

| | |
|---|---|
| **Acute Diseases.** | |
| Typhus | 3 |
| Scarlatina Anginosa | 5 |
| Pneumonia | 6 |
| Rheumatismus Acutus | 2 |
| **Chronic Diseases.** | |
| Tussis | 16 |
| Dyspnœa | 7 |
| Tussis cum Dyspnœa | 14 |
| Catarrhus | 7 |
| Peripneumonia Notha | 2 |
| Phthisis Pulmonalis | 2 |
| Hæmoptoe | 3 |
| Gastrodynia | 3 |
| Enterodynia | 4 |
| Amenorrhœa | 7 |
| Fluor Albus | 5 |
| Cancer Uteri | 1 |
| Epilepsia | 1 |
| Paralysis | 2 |
| Anasarca | 3 |
| Ascites | 1 |
| Hepatitis Chronica | 1 |
| Rheumatismus Chronicus | 20 |
| **Puerperal Diseases.** | |
| Ephemera | 6 |
| Menorrhagia Lochialis | 5 |
| **Infantile Diseases.** | |
| Ophthalmia | 4 |
| Pertussis | 6 |
| Tinea Capitis | 2 |

Since the commencement of the frost, the diseases which usually prevail during the whole of the winter months, but which have been experienced in a very slight degree in consequence of the warm temperature of the air, are much increased in their number, and in the aggravation of their symptoms. Common colds and coughs are become very general; and the more important species of pneumonic disease make their appearance, and in some instances have assumed a very formidable aspect.

Of scarlatina anginosa there have been numerous cases. This disease is usually introduced by chilliness and shivering, and sometimes by a smart rigor, which is succeeded by a sense of fulness about the throat, that in some degree affects the respiration. Deglutition becomes difficult, cult, and, consequently, there is a disinclination to the taking of any kind of nourishment; and in children, this is so great as to prevent their swallowing a necessary quantity of liquid aliment. A scarlet eruption appears very early, which spreads itself over different parts of the surface; but this in a few days disappears, and the fever usually subsides at the same time. In two of the instances referred to in the list, this disease was succeeded by anasarcous swellings, which for a time proved obstinate, but which ultimately yielded to the free use of cathartics and diuretics.

The case of Tinea, which had been troublesome for a considerable time, and had resisted other means, was successfully treated by ung. picis cum sulph. Repeatedly shaving the head contributes to the cure, not only by laying the surface bare for the application of the ointment, but by exciting a gentle stimulus, and promoting the healthy action of cutaneous vessels.

